# The novel estrogen receptor modulator STX attenuates Amyloid-β neurotoxicity in the 5XFAD mouse model of Alzheimer’s disease

**DOI:** 10.1016/j.nbd.2022.105888

**Published:** 2022-10-06

**Authors:** Joseph F. Quinn, Martin J. Kelly, Christopher J. Harris, Wyatt Hack, Nora E. Gray, Veronika Kulik, Zoe Bostick, Barbara H. Brumbach, Philip F. Copenhaver

**Affiliations:** a Department of Neurology, Oregon Health and Science University, Portland, OR, United States of America; b Parkinson’s Disease Research, Education, and Clinical Center, Portland Veterans Affairs Medical Center, Portland, OR, United States of America; c Department of Chemical Physiology and Biochemistry, OHSU, Portland, OR, United States of America; d Department of Cell, Developmental and Cancer Biology, OHSU, Portland, OR, United States of America; e Biostatistics and Design Program, OHSU-PSU School of Public Health, Portland, OR, United States of America

**Keywords:** Amyloid, Alzheimer’s, Estrogen, Mitochondria, Reactive astrocytosis, Microgliosis, Synaptic loss, Behavior

## Abstract

Based on previous evidence that the non-steroidal estrogen receptor modulator STX mitigates the effects of neurotoxic Amyloid-β (Aβ) in vitro, we have evaluated its neuroprotective benefits in a mouse model of Alzheimer’s disease. Cohorts of 5XFAD mice, which begin to accumulate cerebral Aβ at two months of age, were treated with orally-administered STX starting at 6 months of age for two months. After behavioral testing to evaluate cognitive function, biochemical and immunohistochemical assays were used to analyze key markers of mitochondrial function and synaptic integrity. Oral STX treatment attenuated Aβ-associated mitochondrial toxicity and synaptic toxicity in the brain, as previously documented in cultured neurons. STX also moderately improved spatial memory in 5XFAD mice. In addition, STX reduced markers for reactive astrocytosis and microgliosis surrounding amyloid plaques, and also unexpectedly reduced overall levels of cerebral Aβ in the brain. The neuroprotective effects of STX were more robust in females than in males. These results suggest that STX may have therapeutic potential in Alzheimer’s Disease.

## 1. Introduction

Almost two-thirds of Alzheimer’s Disease (AD) patients are women, and the loss of ovarian steroid production at menopause significantly increases their vulnerability to AD pathogenesis (Barron and Pike, 2012; Beam et al., 2018; Nebel et al., 2018). Although the neurotrophic and neuroprotective effects of estrogen are well established (Zarate et al., 2017), clinical trials revealed that different estrogen/progestin formulations caused unacceptable side effects in AD patients (Rapp et al., 2003), including increased risk of thrombosis, hormone-sensitive cancers, and in some cases dementia (Manson et al., 2013; Shumaker et al., 2004). Subsequent trials suggested that initiating hormone replacement therapy at earlier ages (and for shorter periods) could reduce these adverse outcomes (Imtiaz et al., 2017; Kantarci et al., 2018; Song et al., 2020), but even revised protocols of this type still moderately increased the risk for dementia (Maki et al., 2019; Savolainen-Peltonen et al., 2019). Despite these disappointing results, developing compounds that provide the neuroprotective benefits of estrogen signaling without its adverse side effects remains a viable therapeutic strategy for treating AD (Yao and Brinton, 2012).

STX is a selective estrogen receptor modulator (SERM) that might serve this function. Unlike 17β-estradiol (E2) and its analogs, STX does not engage classical estrogen receptors (ERs); rather, STX specifically activates the G protein-coupled membrane estrogen receptor GqMER receptor (Kelly and Ronnekleiv, 2015; Qiu et al., 2003; Qiu et al., 2006), which is expressed by CNS neurons but not by peripheral reproductive organs (Qiu et al., 2008; Roepke et al., 2010). Moreover, orally administered STX readily crosses the blood-brain barrier and recapitulates the beneficial effects of E2 in animal models of menopause (Kaufman et al., 2013; Roepke et al., 2010) and ischemia (Inagaki and Etgen, 2013; Lebesgue et al., 2010). Accordingly, STX can be considered a ‘neuro-SERM’, capable of inducing neuroprotective responses in the brain without the adverse outcomes linked with conventional estrogen replacement therapies in AD patients.

In previous work, we used a variety of cell culture models (including cultured mouse hippocampal neurons) to show that STX treatment attenuated Amyloid-β (Aβ) neurotoxicity in vitro, in part by mitigating Aβ-associated mitochondrial dysfunction and synaptic toxicity (Gray et al., 2016). Accordingly, we have now evaluated the neuroprotective benefits of oral STX in the 5XFAD mouse model of cerebral Aβ deposition (Oakley et al., 2006), which also provides a suitable system for comparing neuroprotective responses in females versus males (Forner et al., 2021; Oblak et al., 2021). Using behavioral tests of spatial memory combined with a subsequent analysis of markers for hippocampal mitochondrial function and synaptic density, we have investigated whether sustained STX treatment can protect against the deleterious effects of neurotoxic Aβ in this well-characterized AD model. We also analyzed the effects of sustained STX administration on amyloid plaque burden and associated reactive gliosis in the brain.

## 2. Methods

### 2.1. Ethics statement and animal use

**Fig. 1.**
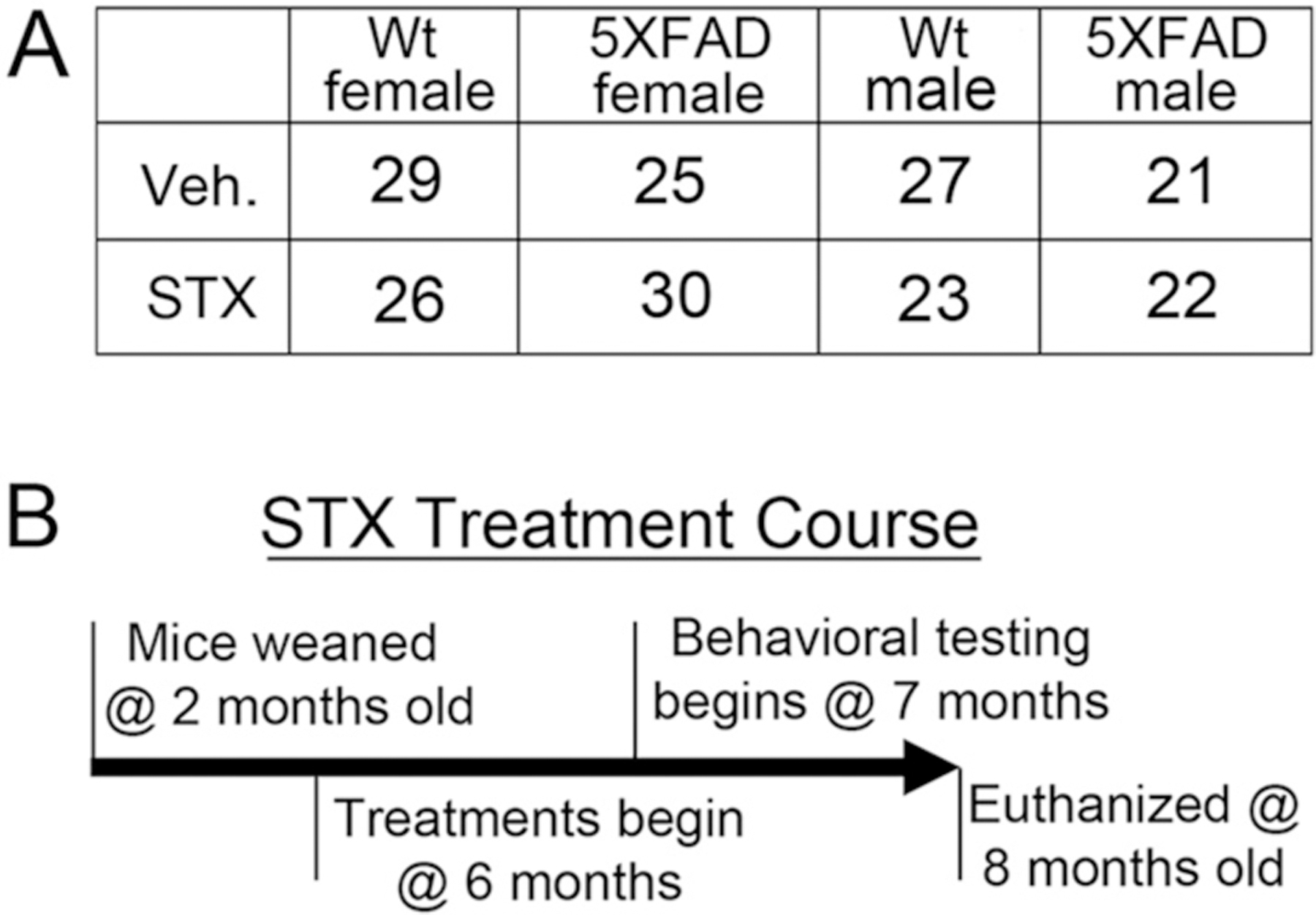
Summary of experimental design used in this analysis. **1A**). Number of mice included for each genotype, sex, and treatment group. **1B**). Treatment regimen used for this study. 5XFAD mice and their wildtype (wt) littermates were weaned at 21 days, genotyped at 2 months of age, and then maintained under normal rearing conditions until 6 months old. Separate groups of females and males of both genotypes were then treated every other day (BID) with either vehicle (Veh) or STX for 2 months, administered orally by gavage. Behavioral testing commenced at 7 months, and the animals were euthanized at 8 months of age to harvest brain tissue for subsequent analysis.

All experiments were conducted in accordance with the NIH Guidelines for the Care and Use of Laboratory Animals and were approved by the institutional Animal Care and Use Committee of the Veteran’s Administration Portland Health Care System (VAPORHCS), under IACUC protocols 3783–20 and 3041–20 (PI- Quinn). 5XFAD and B6SJLF1 mice were purchased from JAX (The Jackson Laboratory, Bar Harbor, ME). 5XFAD mice overexpress human Amyloid Precursor Protein (APP) with three familial Alzheimer’s disease (FAD) mutations [the Swedish (K670N, M671L), Florida (I716V), and London (V7171) mutations] and human PSEN1 with two FAD mutations (M146L and L286V). The mouse colony was maintained by breeding 5XFAD positive male mice with B6SJLF1 females. Wild type littermates were used as controls for each experiment. Animal numbers for each sex and genotype used in this study are summarized in Fig. 1A. All treatment cohorts included both male and female mice, evaluated independently.

### 2.2. Animal rearing and diet

Animals were housed in a climate-controlled facility with a 12-h light/12-h dark cycle. Animals were fed 5LOB rodent diet (Lab Diet, MO) and provided with water and diet *ad libitum*. Mice were weaned at 21 days, genotyped at two months of age, and group-housed (3–4 per cage) until 6 months of age, when experiments commenced.

### 2.3. Treatments and time course of experiments

5XFAD mice begin to accumulate detectable levels of amyloid-β (Aβ) in the brain as early as 2–3 months of age (Kimura and Ohno, 2009), and exhibit substantial numbers of amyloid plaques by 6 months of age (Oakley et al., 2006). Accordingly, mice were randomized into vehicle- or STX-treated groups (21–30 mice per condition; Fig. 1A). For oral treatments, STX was diluted in 50% lactated ringers/50% Pharmasolve (*N*-Methyl-2-pyrrolidone; MilliporeSigma #328634). Beginning at 6 months of age, mice were gavaged with 75 μl of vehicle or vehicle plus STX (18 mg/kg), administered every Monday, Wednesday and Friday for two months (Fig. 1B). Weights were monitored daily to ensure treatment was not detrimental to the animals’ overall health. Behavioral testing was performed on each group from 7 to 8 months of age. At the conclusion of the dosing period, animals were euthanized, and brain tissue was collected for biochemical and immunohistochemical analysis.

### 2.4. Behavioral analysis

#### 2.4.1. Object location memory

The object location memory task is a test of hippocampal-dependent spatial memory. This task was performed in a clear acrylic plastic chamber measuring 39 × 39 cm × 25 cm tall. Testing was conducted over four days. On days 1–2, animals were allowed to explore the empty test area for 10 min each day during a habituation trial. On day 3, animals underwent three training trials. Each training trial lasted 10 min, with a one-hour interval between trials. During training trials, two identical objects were placed in the northwest and northeast corners of the testing area. Two hours after the third training trial, the object in the northeast corner was moved to the southeast corner while the second object remained in the northwest corner. Animals were allowed to explore the area with the object in a novel location for 5 min during a retention trial. Twenty-four hours after the two-hour retention trial, the object that was originally in the northeast corner was then moved to the southwest corner and again the second object remained in the northwest corner. Animals were allowed to explore the testing area for 5 min as part of their 24-h retention trial.

A camera suspended above the testing apparatus was used to record all trials. Distance traveled during the habituation trials was analyzed with ANY-maze behavioral tracking software (Stoelting Co., IL). Training and retention trials were analyzed by investigators blinded to treatment conditions, who recorded the times spent exploring each object. Times during which an animal maintained its nose within 2 cm of the object or kept one paw on the object was counted as time exploring the object. Percent time exploring novel objects was determined by taking the time spent exploring the object in a novel location divided by time exploring both objects.

#### 2.4.2. Morris water maze

The Morris Water Maze (MWM) is another test of hippocampal-dependent spatial memory. This test was conducted using the same apparatus and protocol used in our previous studies (Quinn et al., 2010). A round tank 109 cm in diameter was filled with water (room temperature) that was made opaque with white non-toxic tempura paint. The test consists of 3 phases: visible platform, hidden platform, and probe trials. An adjustable height platform (10 cm in diameter) was placed in the pool during the visible and hidden platform trials. During probe trials, this platform was removed.

During the visible platform trials, the platform was moved in between stages to prevent mice from developing a preference for any single quadrant. The water level was maintained at 1 cm below the lip of the platform. A graduated cylinder wrapped with multicolor tape was also placed on the platform to increase its visibility. Mice were introduced to the tank at pseudo-random drop locations and given 60 s to find the platform. Mice that were unable to find the platform were guided to its location. Mice remained on the platform for 30 s before being removed, dried with a towel, and returned to their cage. Mice were given 12 trials over two days. Each day was divided into a morning and afternoon stage of three trials. Mice were given an inter-trial rest period of 7 min. They were also given at least 2 h rest in between the third and fourth trial of each day. Mice that were unable to consistently find the platform were excluded from further testing.

During the hidden platform phase, the platform was submerged to 1 cm below the water line. Platform location was static for all hidden platform testing. Images were displayed around the room, allowing animals to use spatial learning to memorize the platform location. Mice were introduced into the tank at pseudo-random drop points and given 60 s to find the platform. Mice that failed to find the platform were guided to its location. Once on the platform, mice were removed after 30 s. Mice were tested for thirty trials total over the course of five days: three trials in the morning stage and three trials during the afternoon stage. Mice were allowed to rest for 7 min between each trial, and for 2 h between the third and fourth trial.

Two hours after the last hidden platform trial of each day, mice were given a probe trial. Mice were also given probe trials at 24 h and 72 h after the fifth day of hidden platform trials. During probe trials, the platform was completely submerged to a depth that the mice could not reach. Mice were introduced into the tank at pseudo-random drop sites and allowed to freely swim in the pool for 60 s.

All trials were recorded by a camera suspended over the apparatus and analyzed with ANY-maze Behavioral tracking software. Videos were analyzed for distance traveled (m), duration (s), velocity (m/s) and path efficiency (distance to platform/distance traveled) for the visible and hidden platform trials. During probe trials, videos were analyzed for time in each quadrant (s); percent time in each quadrant (s/60); number of entries to each quadrant; number of entries to previous platform location; and mean distance from previous platform location (m). Percent time in each quadrant over 25% indicated a bias towards the training zone quadrant. All behavioral analyses were conducted by investigators blinded to treatment conditions.

### 2.5. Euthanization and tissue collection

After behavioral analysis, animals were anesthetized with inhalable isoflurane and exsanguinated via cardiac puncture. Brain samples were collected and fixed or frozen. Blood was kept on ice, then spun down in a microcentrifuge at 16,000 x g for 15 min at 4 °C. Serum was collected and frozen at −80 °C. The anterior 3 mm of bilateral prefrontal cortex was dissected and frozen. The right hemisphere was immersion-fixed in 4% paraformaldehyde (PFA; Fisher Scientific, MA) in phosphate buffered saline for histochemical analysis. The contralateral hippocampus, cortex, and deep gray were dissected and frozen on dry ice. Hippocampi were homogenized and mRNA was extracted for quantitative reverse transcription polymerase chain reaction (qRT-PCR), while protein extracts were used for immunoblot assays. The cerebellum was also dissected and frozen for future analysis.

### 2.6. Immunohistochemistry

Right hemispheres were incubated in 4% paraformaldehyde (PFA) for 24 h at room temperature. Following PFA incubation, right hemispheres were incubated for 24 h in PBS, then sequentially incubated in 15% and 30% sucrose solutions before being frozen at −80 °C for sectioning. Frozen coronal sections (40 μm) were cut on a freezing microtome. Floating sections were then stored in sectioning solution (10% glycerol, 10% methanol in TBS) before subsequent processing.

Sections were placed in tris-buffered saline (TBS) for 5 min, Quench (30% hydrogen peroxide, 10% methanol) for 2 min, rinsed in TBS for 5 min, and then incubated with agitation in blocking buffer (100 mM TBS, pH 8.0, 2 mg/ml bovine serum albumin, 2% horse serum, 0.5% Triton X-100) for 3 h. Sections were then incubated overnight at room temperature with one of the following primary antibodies that we validated in our previous studies (Quinn et al., 2007), diluted 1:1000 in blocking buffer: anti-Aβ polyclonal antibody (Thermo Scientific, MA #44–136); anti-Glial Fibrillary Acidic Protein (GFAP; Biomedical Technologies, MA #BT-575); or with biotinylated *Griffonia simplicifolia* Lectin I (GSL 1; Vector Labs, CA #B-1205).

The following day, sections were washed three times in TBS (5 min each wash). For detecting primary antibodies specific for pan-Aβ and GFAP, sections were then incubated for 2 h in biotinylated goat-anti-rabbit secondary antibodies (1:200; Vector laboratories; BA-1000). Sections were subsequently washed twice in TBS for 5 min and once in PBS for 5 min. They were then incubated for 2 h with an avidin-linked peroxidase complex (AB-HRP kit #PK-4000, Vector Laboratories), washed three times in PBS for 5 min, and developed with diaminobenzidine (Sigma-Aldrich #D4418) plus H_2_O_2_ in PBS, as previously described (Harris et al., 2014). Sections were then washed three times in PBS for 5 min and placed on Superfrost Plus Coverglass slides (Fisher Scientific, MA). The sections on slides were dehydrated in 50%, 50%, 75%, 95%, 100%, 100% and 100% EtOH for ten min each, incubated in Citrisolv (three times for 10 min), and mounted with Permount (Fisher Scientific, Pittsburg, PA). Pathology was quantified in three coronal sections representing regions of anterior, middle and posterior hippocampus and cortex from each mouse. Hippocampal and cortical areas were traced using a computerized stage and stereo investigator software (Image J, Wayne Rasband, NIH, USA). The extent of Aβ pathology was expressed as percentage of hippocampus or cortex that was occupied by detectable immunoreactive staining. Mean values for each parameter were calculated from at least three sections per animal.

### 2.7. Quantitative Reverse Transcription PCR

Hippocampal tissue was homogenized in Tri-Reagent (Sigma-Aldrich, MO). BAN phase separation reagent (Molecular Research Center, Inc., OH) was then added, and the samples were centrifuged at 16,000 rcf for 15 min at 4 °C. The top layer of each sample was transferred into a new microfuge tube with an equal volume of isopropanol and centrifuged at 16,000 rcf for 8 min at 4 °C. The supernatant was mixed with 75% ethanol, centrifuged for 5 min at 16,000 rcf, and the pellet was resuspended in DiH_2_O. cDNA was generated from the samples using Superscript III First-Strand Synthesis System (Thermo Fisher, MA), as per the manufacturer’s protocol.

Relative gene expression was determined using Taqman Gene Expression Master Mix (Thermo Fisher, MA) and commercially available TaqMan primers (Thermo Fisher, MA) on an Applied Biosystems StepOnePlus Real-Time PCR System (Thermo Fisher, MA). All primers were normalized to glyceraldehyde-3phosphate dehydrogenase (GAPDH; Hs02758991_g1). Other primers used were specific for post-synaptic density protein 95 (PSD95 – Mm00492193_m1), Synaptophysin (Mm00436850_m1), mitochondrially encoded cytochrome *b* (Mt-CYB; Hs02596867_s1), LOC101928524 (Mt-ATP6; Hs02596862_g1), mitochondrially encoded cytochrome *c* oxidase 1 (Mt-CO1; Hs02596864_g1) and mitochondrially encoded NADH dehydrogenase 1 (Mt-ND1; Hs02596873_s1). Relative expression was determined using the delta-delta Ct method.

### 2.8. Protein extraction

Protein was also extracted from the samples using the bottom layer of each sample after the BAN phase separation described above. This layer was incubated with 500 μl acetone for 10 min at room temperature, then centrifuged at 12,000 rcf at 4 °C for 10 min. The supernatants were discarded, and the pellets sonicated in 1000 μl of “protein wash 1” (95% ethanol, 2.865% guanadine, 2.5% glycerol, and 2.5% water). After incubation at room temperature for 10 min, the samples were centrifuged at 8000 rcf at 4 °C for 10 min. The supernatants were again discarded, and the samples were sonicated in 1000 μl “protein wash 1” for two more cycles. The supernatants were discarded, and the remaining samples were sonicated in 1000 μl of “protein wash 2” (95% ethanol, 2.5% glycerol, 2.5% water). After 10 min incubation at room temperature, the samples were centrifuged at 8000 rcf at 4 °C for 5 min. The supernatants were discarded, and any residual supernatant allowed to evaporate at room temperature. The final pellets were re-suspended in 250 μl 1% SDS+ protease and phosphatase inhibitor cocktail (Thermo Fisher, MA).

### 2.9. Western blots

Lysates of dissected prefrontal cortex from 5XFAD and wild type littermate mice were prepared as described above and diluted in lysis buffer: phosphate-buffered saline (PBS) plus 1% NP-40, 1× Protease Inhibitor Cocktail (Sigma-Aldrich #P8340), 1× Antipain Dihydrochloride (Sigma-Aldrich #A6191), and 1× PhosSTOP (Roche #4906845001). The samples were then denatured for 5 min at 50 °C (for subsequent immunoblotting with antibodies against OXPHOS proteins) or 95 °C (for immunoblotting with antibodies against synaptic markers). After brief centrifugation, samples were separated by electrophoresis in 26-well Criterion XT 12% Bis-Tris polyacrylamide gels (BioRad #3450119), run in triplicate to provide technical replicates (1 μg total protein per lane). In addition, replicate lysates prepared from cortical tissue of a wild type control mouse were included in each gel (indicated as ‘cntrl ctx’ in each immunoblot) to normalize for differences in transfer and imaging conditions. Each gel included samples from a combination of different treatment groups (male versus female; wt versus 5XFAD; vehicle-treated versus STX-treated) to allow for quantitative comparisons of relative protein expression. A total of 12 gels with varying sets of different treatment groups were generated for each experiment (Suppl. Fig. S1 & S2), and comparisons between treatment groups in the same immunoblots were analyzed by an investigator blinded to the treatments. Electrophoresed samples were then transferred to low-fluorescence PVDF membranes (Immun-Blot; BioRad #162–0263) using a semi-dry transfer apparatus in Tris/Glycine buffer. The gels were stained with GelCode Blue (Thermo-Fisher # 24590) to monitor transfer efficiency, and the membranes were rinsed repeatedly in blocking buffer (TBST plus 5% Blotto nonfat dry milk; Rockland #B501–0500). Membranes were subsequently incubated overnight at 4 °C with primary antibodies, diluted in 1× Tris-buffered saline plus 0.1% Tween 20 (TBST). The membranes were then rinsed in 1× TBST for 5 min with gentle rocking, and incubated with appropriate secondary antibodies for 30 min at room temperature. After rinsing in 1× TBST for 5 min, the membranes were incubated with enhanced chemiluminescence substrate (ECL; SuperSignal West Pico PLUS; Thermo-Fisher #34579) and visualized sequentially on X-ray film and on a c600 Azure imager (with the lowest sensitivity setting). The digital Azure images were subsequently analyzed using AzureSpot software and analyzed in Excel. The following primary antibodies against synaptic proteins were used in this analysis: rabbit-anti-PSD95 (Cell Signaling Technologies #3450; 1:1000) and Rabbit-anti-synaptophysin (Cell Signaling Technologies #36406; 1:1000), which were applied and imaged simultaneously. The membranes were then stripped and labeled with mouse-anti-GAPDH (R&D # 5718; 0.5 μg/ml) as a loading control. To detect components of the oxidative/phosphorylation pathway, we used the total OXPHOS Rodent WB Antibody Cocktail (Abcam #ab110413 1.5 μg/ml), which includes antibodies against the following proteins: Complex I subunit NDUFB8 (NADH dehydrogenase [ubiquinone] 1 beta subcomplex subunit 8; 20 kDa); Complex II SDHB (Succinate dehydrogenase [ubiquinone] iron‑sulfur subunit, 30 kDa); Complex III UQCRC2 (ubiquinol-cytochrome C reductase Core Protein 2; 48 kDa); Complex IV MTCO1 (Cytochrome C oxidase subunit I; 40 kDa); and Complex V ATP5A (ATP synthase F1 subunit alpha; 55 kDa). However, in lysates extracted with Tri-Reagent, MTCO1 was not readily detected and was not included in the current analysis. The membranes were then stripped and labeled with mouse-anti-GAPDH as a loading control. To analyze protein expression levels, background subtraction was first performed for each band per sample using the ‘rolling ball’ function in AzureSpot software, and relative values were determined as ratios to GAPDH intensities for each band. To compare samples across multiple immunoblots, intensity values were normalized against the average values obtained from identical lysate samples of wild type control mouse cortex, run in triplicate on each gel. Quantification was performed blind to genotype and treatment conditions.

### 2.10. Power analysis

In previous studies testing the benefit of α-lipoic acid (ALA) in AD mouse models (Quinn et al., 2007), we found that a significant difference would be observable with a minimum of 8 mice per diet group. Using percent dwell time in the target platform quadrant during the probe trial as our outcome measure in MWM tests, we compared the effects of ALA in transgenic mice (*N* = 15, 9 ALA diet, 6 control diet) versus controls (*N* = 17, 9 ALA diet, 8 control diet). In two-way ANOVA tests, we found a significant effect caused by ALA (F_1,28_ = 4.93, *p =* 0.035) across the entire cohort, with an additional diet-dependent effect in the transgenic mice (F_1,28_ = 9.73, *p =* 0.004), corresponding to an on-treatment effect size of Cohen’s guidelines (d = 1.47). Even if we assumed the effect of ALA was pervasive within transgenic mice, a significant difference would still be observable with a minimum of 8 mice per group at a two-tailed significance level of α = 0.05 with 90% power.

### 2.11. Statistics

The distributions for all continuous outcome variables were assessed and we found that all variables violated assumptions of normality. Therefore, we used the nonparametric Kruskal-Wallis test to test for significant differences between groups. Dunn’s Multiple Comparison Test (with Bonferroni corrections) was used for post hoc pairwise comparisons. Box and whisker plots were used to graphically display the data, designating the interquartile range (IQR) and median values for each group. Effects were considered significant at *p* ≤ 0.05. Statistically significant differences are indicated in each figure via the following convention: *p < 0.05, ^**^p < 0.01, ^***^p < 0.001 ^****^p < 0.0001.

## 3. Results

### 3.1. Extended treatment with oral STX was well tolerated by wild type and 5XFAD mice

In previous studies, short-term oral treatment with STX in mouse models of both ischemia and menopause had no adverse side effects and was found to provide many of the protective benefits of E2 in the brain (Inagaki and Etgen, 2013; Kaufman et al., 2013; Lebesgue et al., 2010; Roepke et al., 2010). To investigate the neuroprotective benefits of oral STX in an AD model, we treated matched cohorts of 5XFAD mice for 2 months starting at 6 months of age (Fig. 1), when substantial numbers of amyloid plaques (and associated reactive gliosis) are readily detectable in the brain (Oakley et al., 2006). We found that chronic administration of STX was well tolerated by both wild type and 5XFAD mice over the course of our two-month study. We observed no evidence of abnormal weight loss in either males or females (+2.05 g in STX-treated versus +2.25 g in vehicle-treated groups), nor did we observe any excess mortality associated with our treatment protocol: over the course of this eight-month experiment, we documented 8 deaths in STX-treated groups versus 9 deaths in vehicle-treated groups of undetermined cause (a rate of 8.5%), comparable to low death rates seen in previous studies. Likewise, we did not detect any signs of hyperactivity or excitotoxicity associated with STX treatments, which have been observed in some experiments using 5XFAD mice (Flanigan et al., 2014; Oblak et al., 2021).

### 3.2. Protective effects of STX effects on Aβ-associated mitochondrial toxicity

**Fig. 2.**
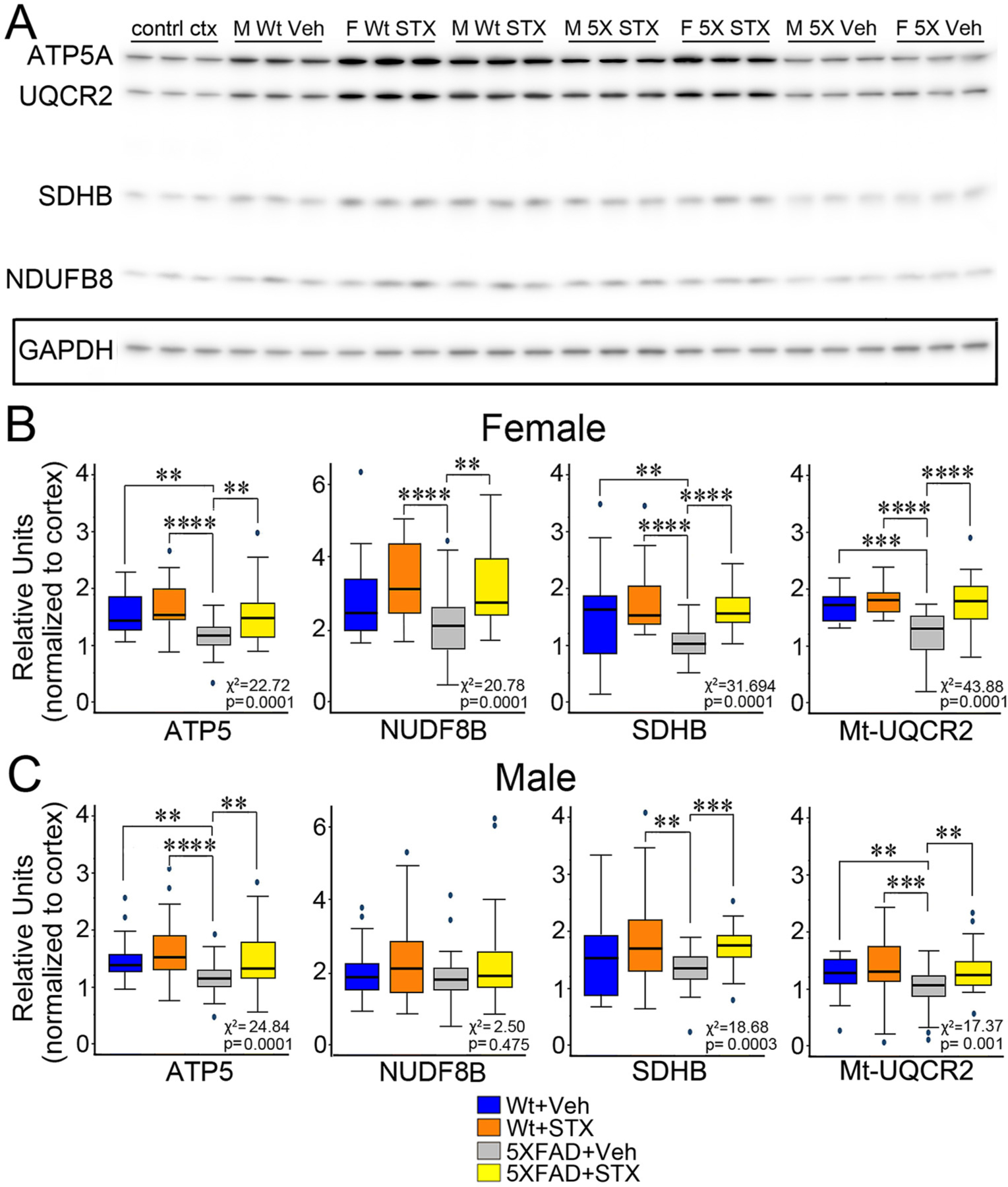
Oral STX treatment mitigated the reduction in Electron Transport Chain (ETC) protein expression in the hippocampus of 5XFAD mice. **2A)**: Montage of representative western blots of protein lysates prepared from the hippocampi of each treatment group at the conclusion of behavioral testing (at 8 months of age). Samples were run in replicate and normalized by comparison with identical samples of control cortical lysates included on each gel (not shown). Antibodies against the following ETC proteins were included in the total OXPHOS Rodent Western Blot Antibody Cocktail (Abcam): ATP5A (Complex V ATP synthase F1 subunit alpha; 55 kDa); UQCR2 (Complex III ubiquinol-cytochrome C reductase Core Protein 2; 48 kDa); SDHB (Complex II Succinate dehydrogenase [ubiquinone] iron‑sulfur subunit, 30 kDa); and NDUFB8 (Complex I subunit NADH dehydrogenase [ubiquinone] 1 beta subcomplex subunit 8; 20 kDa). Westerns were subsequently stripped and re-probed with anti-GAPDH (37 kDa) as a loading control (boxed row). **2B)**. Comparison of OXPHOS protein expression levels in 5XFAD females and wildtype (wt) female littermates that had been treated with vehicle or STX for two months. Values for each treatment group (indicated in relative units) were first calculated as a ratio with GAPDH per lane and then normalized against replicate samples of control mouse cortex (to control for differences in loading and exposure between gels). STX treatment significantly improved the expression of all four OXPHOS proteins in 5XFAD females, maintaining levels that were similar to vehicle-treated wt females, and moderately increased OXPHOS proteins in wt females. **2C**). STX also attenuated the reduction in OXPHOS proteins in 5XFAD males. Results from the Kruskal-Wallis tests are reported in each fig. *N* ≥ 10. Dunn’s pairwise comparisons are denoted with the following: ^**^p < 0.01; ^***^p < 0.001; ^****^p < 0.0001.

In previous work, we showed that STX improved key aspects of mitochondrial function in cultured hippocampal neurons from the Tg2576 mouse model of amyloid pathology, including enhanced expression of genes encoding proteins in the electron transport chain (ETC) (Gray et al., 2016). To investigate whether this beneficial effect could be recapitulated in intact animals, we treated 5XFAD mice and their wild type littermates with either vehicle or STX for two months. After conducting behavioral studies (described below), we prepared lysates from both hippocampal and prefrontal cortical brain regions for an analysis of ETC protein expression, using our quantitative immunoblotting methods (Ramaker et al., 2016). An example of one of these immunoblots (illustrating the different treatment groups) is shown in Fig. 2A, which was visualized using AzureSpot software and analyzed in Excel. After applying a Kruskal-Wallis test to assess whether group status was significantly associated with the outcome, we used Dunn’s test to determine statistical significance for pairwise comparisons.

As shown in Fig. 2B, we found that the relative expression of several key ETC proteins was significantly associated with group status in females: ATP5 (nuclear-encoded ATP synthase F1 subunit alpha; *χ*^*2*^ *=* 22.72, *p =* 0.0001), SDHB (nuclear-encoded Succinate dehydrogenase [ubiquinone] iron‑sulfur subunit; *χ*^*2*^ = 31.69, p = 0.0001), Mt-UQCR2 (mitochondrially encoded ubiquinol-cytochrome C reductase Core Protein 2; *χ*^*2*^ = 43.88, p = 0.0001), and NUDF8B (nuclear-encoded NADH dehydrogenase beta sub-complex subunit 8; *χ*^*2*^ *=* 20.78, p = 0.0001). Using Dunn’s pairwise comparisons, we found that relative expression was markedly reduced in vehicle-treated 5XFAD females (gray boxes) compared to vehicle-treated wild type female littermates (blue boxes), including ATP5A (z = 3.3, *p* < 0.01), SDHB (z = 3.4, p < 0.01), and Mt-UQCR2 (z = 4.0, *p* < 0.001). In contrast, STX treatment mitigated this pattern of reduced expression in 5XFAD females (yellow boxes) compared to vehicle-treated 5XFAD females (gray boxes) for ATP5 (z = −3.1, p < 0.01), SDHB (z = −4.6, *p* < 0.0001), and Mt-UQCR2 (z = −5.3, p < 0.0001); thus maintaining levels that were similar to vehicle-treated wild type females. Likewise, the expression of NDUFB8 was moderately reduced in 5XFAD females (not statistically significant), while STX treatment did significantly improve its expression, compared to vehicle-treated 5XFAD females (z = −3.5, p < 0.01). Similar effects were seen in males (Fig. 2C): ATP5 (χ2 = 24.84, p = 0.0001), SDHB (χ2 = 18.68, p = 0.0003), Mt-UQCR2 (χ2 = 17.37, p = 0.001), and NUDF8B (χ2 = 2.50, p = 0.475). The expression levels of both ATP5 (z = 3.4, p < 0.01) and Mt-UQCR2 (z = 2.9, p < 0.01) were significantly reduced in vehicle-treated 5XFAD animals (compared to wild type males). Notably, they were protected by STX treatment in ATP5 (z = −2.9, p < 0.01), SDHB (z = −3.8, p < 0.001), and Mt-UQCR2 (z = −3.0, p < 0.01), which also significantly improved expression of in 5XFAD males. STX treatment also moderately increased the expression levels of several ETC proteins in wild type females and males (compared to vehicle-treated animals), although these effects did not reach significance.

**Fig. 3.**
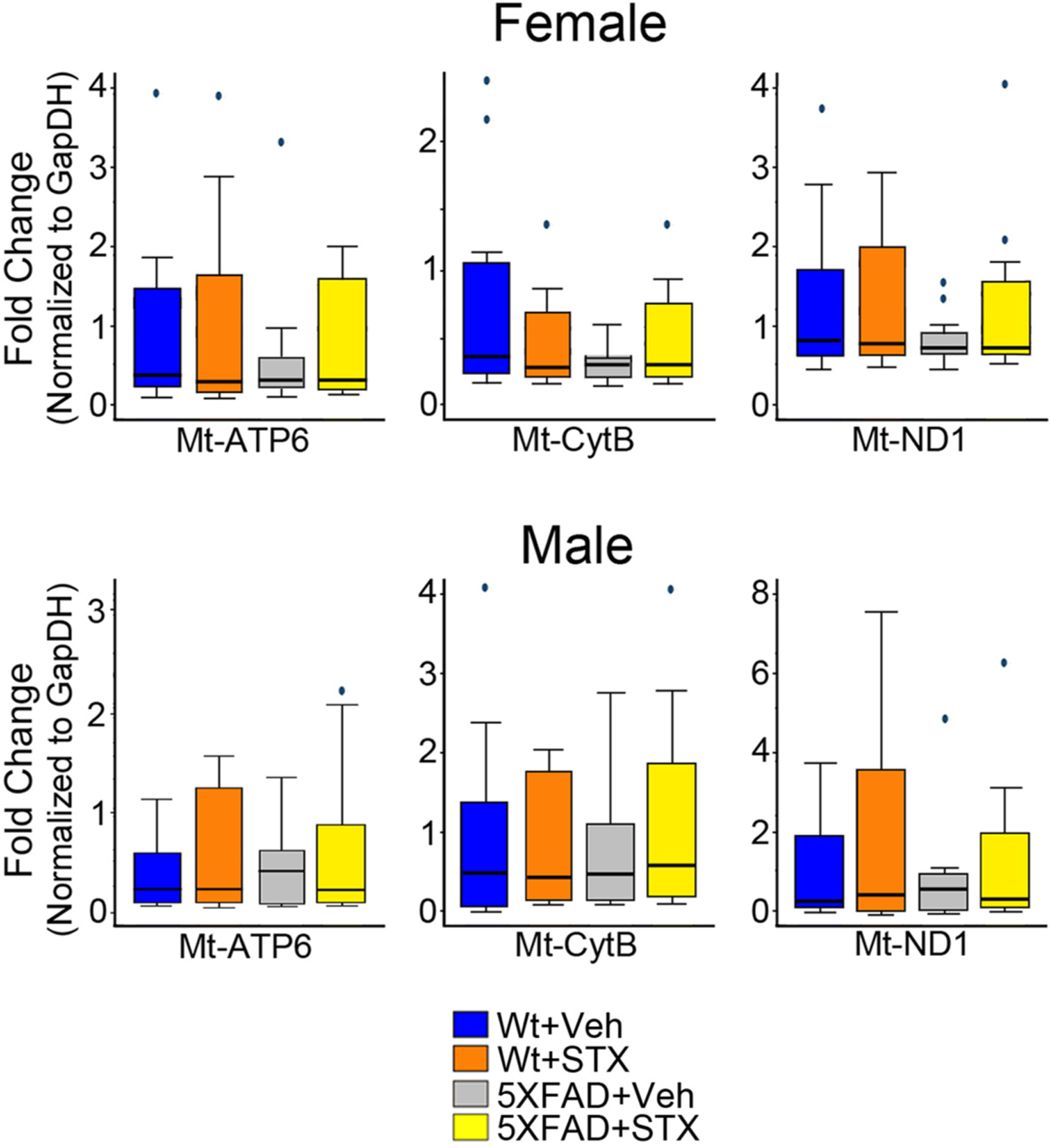
Oral STX treatment attenuated the loss of ETC gene expression in the hippocampus of 5XFAD females. Hippocampal lysates from animals that had been treated for 2 months with vehicle or STX were used to evaluate the relative expression of the following mitochondrial electron transport genes by qRT-PCR: Mt-ATP6 (mitochondrially encoded ATP synthase), Mt-CYB (mitochondrially encoded cytochrome B), Mt-ND1 (mitochondrially encoded NADH dehydrogenase 1), and Mt-CO1 (mitochondrially encoded cytochrome *c* oxidase 1).

As a complementary method, we also examined the effect of STX treatment on the expression of ETC genes in hippocampal lysates (Fig. 3). We observed that mRNA levels encoding several ETC proteins trended lower in some vehicle-treated female 5XFAD mice compared to vehicle-treated wild type females, including Mt-ATP6 (mitochondrially encoded ATP synthase), Mt-CYB (mitochondrially encoded cytochrome B), and Mt-ND1 (mitochondrially encoded NADH dehydrogenase 1), an effect that partially offset by STX treatment. However, in contrast to our analysis of mitochondrial protein expression (Fig. 2), the relative differences in mRNA expression for ETC genes between vehicle-treated wild type versus 5XFAD mice were unexpectedly subtle. In addition, there was substantial variability within the different treatment groups, and the results of this assay did not reach statistical significance. Nevertheless, in combination with our immunoblot analysis, these results suggest that sustained oral treatment with STX protects against the loss of normal ETC gene expression in 5XFAD mice, a beneficial effect that was more pronounced in females than males.

Although STX treatment supported wild type expression levels in some 5XFAD animals, these differences did not reach significance (*p* > 0.05) in this assay (Kruskal-Wallis tests). *N* ≥ 10.

### 3.3. Protective effects of STX on Aβ-associated synaptotoxicity

**Fig. 4.**
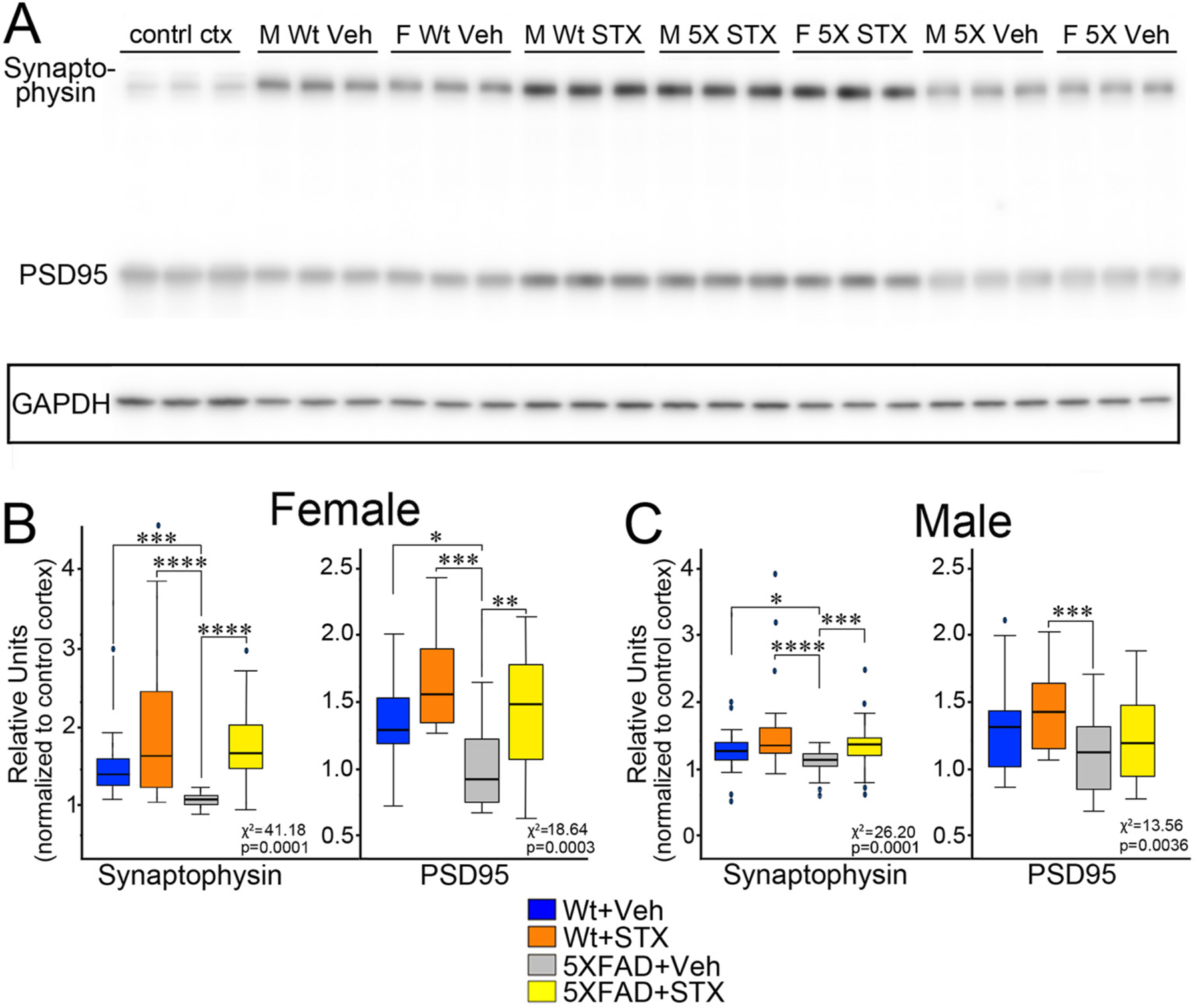
Oral STX treatment mitigated the reduction in synaptic protein expression in the hippocampus 5XFAD mice. **4A)**. Montage of representative western blots of protein lysates prepared from the hippocampi of each treatment group at the conclusion of behavioral testing (at 8 months of age). As in Fig. 3, samples were normalized by comparison with identical control cortical lysates included on each gel (not shown). Western blots were labeled with rabbit antibodies against the postsynaptic protein PSD95 (95 kDa) and the presynaptic protein synaptophysin (38 kDa) that were applied and visualized simultaneously; the blots were then stripped re-probed with anti-GAPDH (boxed row). **4B**). Comparison of synaptic protein expression levels in 5XFAD females versus wildtype (wt) female littermates treated with vehicle or STX for two months. STX treatment significantly improved the expression of both synaptophysin and PSD95 in 5XFAD females, and moderately increased their expression in wt littermate females. **4C**). STX treatment also mitigated the reduction in synaptophysin expression seen in 5XFAD males and moderately improved PSD95 levels. Results from the Kruskal-Wallis tests are reported in each fig. *N* ≥ 10. Dunn’s pairwise comparisons are denoted with the following: *p < 0.05; ^**^p < 0.01; ^***^p < 0.001; ^****^p < 0.0001.

To examine the potential benefits of STX treatment in maintaining synaptic integrity, we also used our quantitative immunoblotting protocols to analyze the expression of the pre-synaptic marker synaptophysin and the post-synaptic marker PSD95 in hippocampal lysates from each treatment group. In females, relative expression levels of both synaptic markers was significantly associated with group status: for synaptophysin, *χ*^*2*^ *=* 41.18, *p =* 0.0001; for PSD95, *χ*^*2*^ *=* 18.64, *p* = 0.0003. As shown in Fig. 4A–B, 5XFAD females (gray boxes) showed significant reductions in both synaptic proteins compared to vehicle-treated wild type females (blue boxes): for synaptophysin: z = 4.1, *p* < 0.001; for PSD95: z = 2.5, *p* < 0.05. Notably, STX treatment prevented this deleterious effect in 5XFAD females (yellow boxes; for synaptophysin: z = −6.2, *p* < 0.0001; for PSD95: z = −3.3, *p* < 0.01) and slightly improved their expression in wild type females (not statistically significant). Likewise, we observed significantly reduced synaptophysin in 5XFAD males (gray boxes) compared to vehicle-treated wild type males (blue boxes; z = 2.7, *p* < 0.05), an effect that was prevented by STX treatment (Fig. 4C). A similar trend was seen for PSD95, whereby expression levels in STX-treated 5XFAD males (yellow boxes) was not significantly different from vehicle-treated wild type males (blue boxes; z = 1.1, *p* = 0.82).

**Fig. 5.**
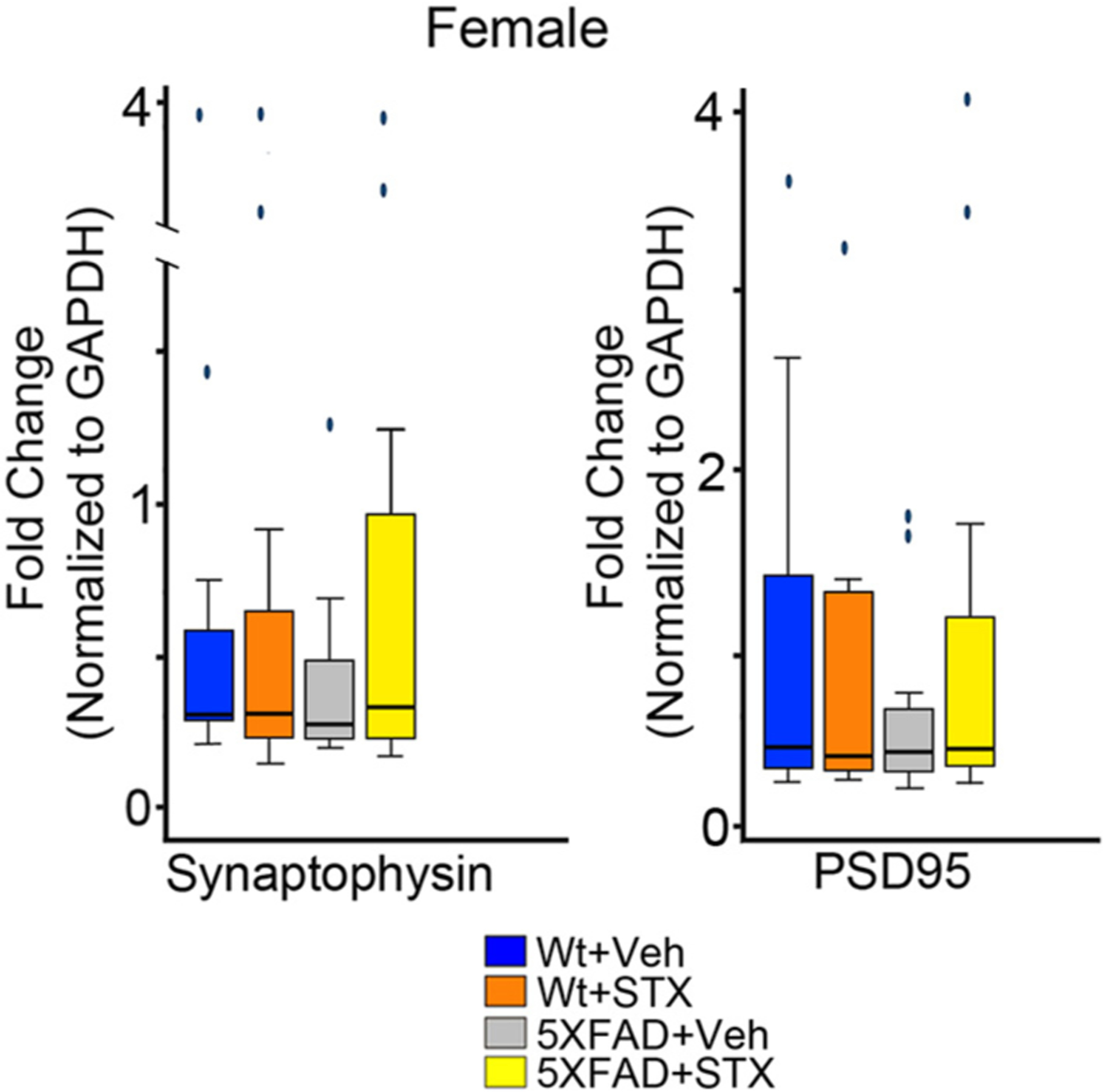
Effects of oral STX treatment on synaptic gene expression in the hippocampus of 5XFAD females. Hippocampal lysates from animals treated with vehicle or STX were used to evaluate the relative expression of mRNA encoding the pre-synaptic marker synaptophysin and the post-synaptic marker PSD95. STX treatment mitigated the loss of normal expression levels in 5XFAD females. Results from Kruskal-Wallis tests are reported in each fig. N ≥ 10.

By comparison, when we examined the expression of synaptophysin and PSD95 gene expression using qRT-PCR methods, we did not detect statistically significant effects of STX treatment in either wild type or 5XFAD groups. Although we observed that relative mRNA levels trended lower in some female 5XFAD mice (compared to wild type controls), an effect that was offset by oral STX, there was considerable variability within the different treatment groups, and these effects did not reach statistical significance (Fig. 5). Likewise, male 5XFAD mice in this study did not exhibit reductions in synaptic gene expression compared to wild type males, and correspondingly, STX did not noticeably improve their expression in either genotype (not shown). Nevertheless, our results suggest that oral STX also protects against the loss of key synaptic proteins (as markers for Aβ-associated synaptotoxicity), although again this beneficial effect was more consistent in females than males.

### 3.4. Effects of STX on Aβ-associated deficits in hippocampal dependent memory

**Fig. 6.**
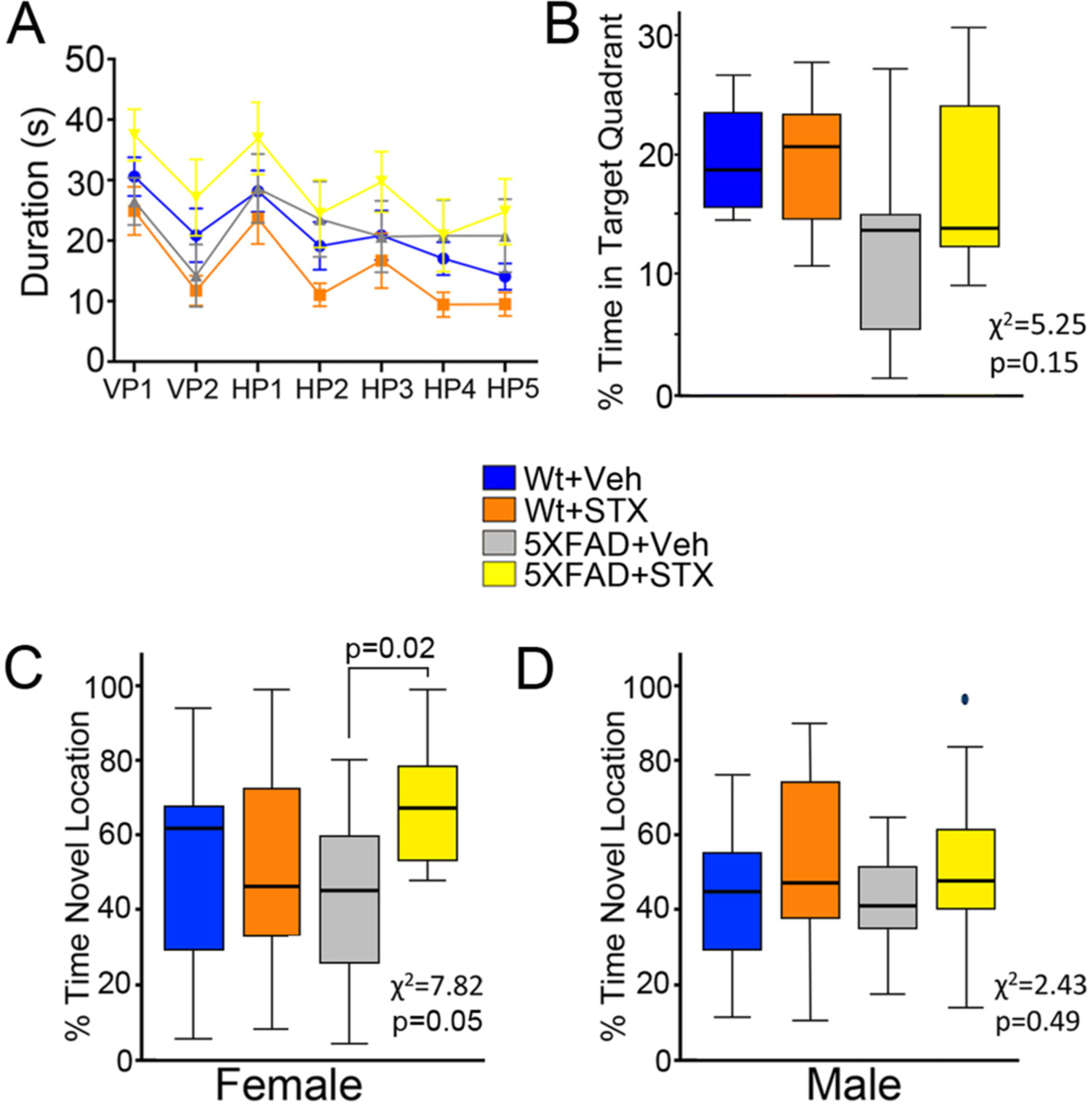
Oral STX treatment protected against the decline in hippocampal-dependent memory in 5XFAD female mice. Animals that had been orally treated with vehicle or STX for 2 months (from 6 to 8 months of age) were analyzed for cognitive responses using the Morris Water Maze (MWM) test and novel object location memory test. **6A).** In the MWM test, STX treatment did not significantly alter the performance of wild type or 5XFAD females in either the visible or hidden platform assays. **6B).** Vehicle-treated 5XFAD females exhibited moderately reduced performance in the probe test, which was mitigated by STX. **6C**). In the object location memory (OLM) test, vehicle-treated 5XFAD females spent significantly less time exploring the object in the novel location (compared with vehicle-treated wild type females), whereas STX treatment prevented this loss in performance. **6D**). STX moderately improved spatial memory performance in both wild type and 5XFAD males (not significant. Results from the Kruskal-Wallis tests are reported in each fig. *N* ≥ 13 for OLM tests; *N* ≥ 8 for MWM tests. In C, *p < 0.05 (Dunn’s pairwise comparison).

We also examined the potential benefits of oral STX on hippocampal-dependent spatial memory. Several groups have previously reported behavioral deficits in aged 5XFAD mice using Morris Water Maze (MWM) tests (Gu et al., 2018; Kang et al., 2018; Ullah et al., 2021), although these effects tend to be more subtle than in other behavioral assays. In female mice that were tested at 7–8 months of age, we did not detect any genotype differences between vehicle-treated 5XFAD and wild type animals in either the visible or hidden platform assays (Fig. 6A), nor did STX treatment affect performance of female mice in either phase. However, in the probe test (Fig. 6B), we noted spatial memory was moderately reduced in vehicle-treated female 5XFAD mice (gray box) compared to vehicle-treated wild type females (blue box), a trend that appeared to be mitigated by STX treatment (yellow box). In contrast, we detected no significant effects of 5XFAD genotype or of STX treatment in any phase of the MWM test in male mice (not shown).

As a complementary approach, we also used the object location memory assay of hippocampal-dependent spatial memory (Creighton et al., 2019; de Pins et al., 2019; Gray et al., 2018). Similar to previous reports using this assay (de Pins et al., 2019; Tible et al., 2019), we found a statistically significant association between time spent exploring the novel location and group status (*χ*^*2*^ = 7.82, *p* = 0.05). We also noted that vehicle-treated 5XFAD females tended to spend less time exploring the object in the novel location when compared with vehicle-treated wild type mice (Fig. 6C), although this trend did not reach statistical significance. In contrast, female 5XFAD mice treated for 2 months with STX showed improved spatial memory performance compared to vehicle-treated 5XFAD females (z = −2.8, *p* < 0.05). Likewise in males, STX treatment produced a modest improvement in spatial memory performance in both wild type and 5XFAD males, but this trend was not significant in either genotype (Fig. 6D). These behavioral assays indicated that STX treatment was well tolerated and had moderate beneficial effects on cognitive performance that were more apparent in female 5XFAD mice. Our results are consistent with recent comprehensive analyses of this mouse model, indicating that AD-associated pathology is more aggressive in 5XFAD females than males (Forner et al., 2021; Oblak et al., 2021).

### 3.5. STX effects on hippocampal Aβ levels and Aβ-associated reactive gliosis

**Fig. 7.**
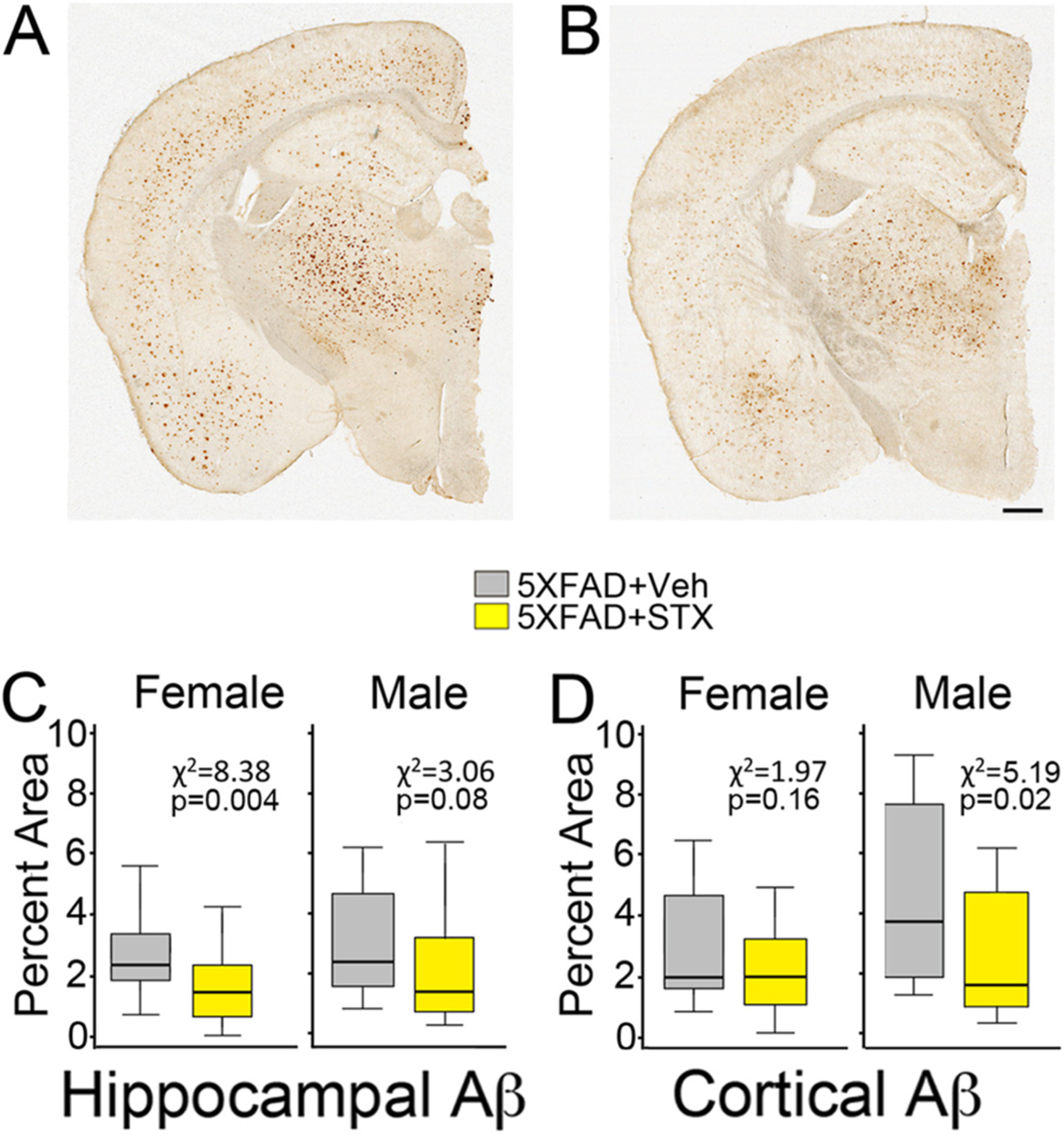
Oral STX treatment reduced Aβ plaque burden in 5XFAD female mice. Brain slices from 5XFAD animals that had been orally treated with vehicle or STX for 2 months (from 6 to 8 months of age) were immunostained with anti-Aβ antibodies. Aβ pathology was expressed as a percentage of the hippocampus and cortex occupied by detectable immunoreactive staining. Scale bar = 1 mm. **7A**). Brain section from an 8 month old vehicle-treated 5XFAD female contained abundant amyloid plaques throughout the hippocampus (h) and cortex (c). **7B**). Section from an 8 month old STX-treated 5XFAD female contained substantially less Aβ pathology in all brain regions. **7C**). lower plaque densities within hippocampal regions of 5XFAD females, with a more moderate reduction in males (consistent with our other assays). **7D**) A similar trend was apparent in the cerebral cortex, although this effect did not reach statistical significance. Plaque density (calculated as percentage of brain area occupied by detectable immunoreactive staining). *N* ≥ 17. Results from Kruskal-Wallis tests with *p* values are shown in each panel.

As noted above, we initiated STX treatment in 5XFAD mice at 6 months of age, when substantial numbers of amyloid plaques and associated reactive gliosis are already detectable in the brains of these mice (Oakley et al., 2006). Although the engagement of GqMER signaling by STX is not predicted to affect the generation or accumulation of Aβ directly, we used our published methods to analyze amyloid plaque distributions after the completion of our behavioral analyses. Unexpectedly, we found that Aβ burden was noticeably reduced in brain sections prepared from STX-treated 5XFAD animals, compared to vehicle-treated 5XFAD animals (Fig. 7A–B). Higher magnification views of amyloid plaques are shown in Supplemental Fig. 3A and C. In particular, STX treatment resulted in significantly lower plaque densities within hippocampal regions of 5XFAD females (Fig. 7C; *χ*^*2*^ = 8.38, *p* = 0.004), with a more modest reduction in males (consistent with our other assays). Likewise, Aβ burden was reduced in the cerebral cortex of STX-treated 5XFAD males (Fig. 7D; *χ*^*2*^ *=* 5.19, *p =* 0.02), with a more subtle response in females (not significant).

**Fig. 8.**
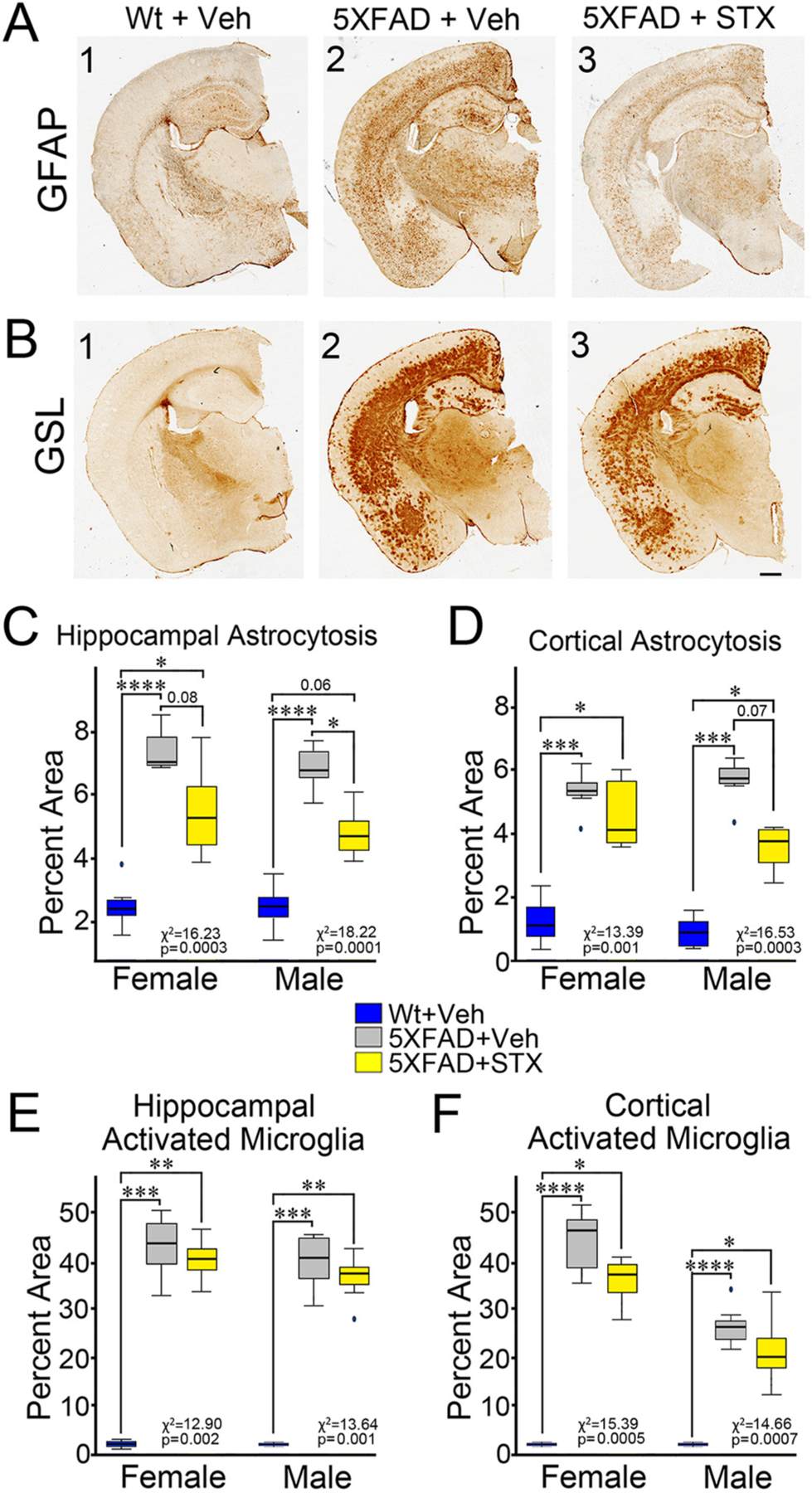
Oral STX treatment reduced markers of reactive gliosis in both male and female 5XFAD mice. **8A**). Brain slices from 5XFAD animals treated with vehicle or STX from 6 to 8 months of age were immunostained with antibodies against GFAP (a marker for reactive astrocytes). **8B**). Brain sections from 8 month old mice labeled with the GSL (a marker for activated microglia. Scale bar = 1 mm. **8C**). Quantification of reactive astrocytosis (based on anti-GFAP staining) in hippocampal regions of females and males. **8D**). Quantification of reactive astrocytosis (based on anti-GFAP staining) in cortical regions of females and males. **8E-F**) Quantification of activated microglia (based on biotinylated GSL labeling) in different brain regions of females and males. *N* ≥ 6. Results from the Kruskal-Wallis tests are reported in each figure. Dunn’s pairwise comparisons are denoted with the following: *p < 0.05; ^**^p < 0.01; ^***^p < 0.001; ^****^p < 0.0001.

We also examined whether STX modified the well-documented patterns of reactive gliosis that accompanies amyloid accumulation in 5XFAD mice (Forner et al., 2021; Zhang et al., 2021). To analyze the distribution of reactive astrocytes, we immunostained brain sections with antibodies against GFAP (Eng and Ghirnikar, 1994; Yang and Wang, 2015), which is strongly associated with amyloid plaque load in AD (Kamphuis et al., 2014). Compared to vehicle-treated wild type mice (Fig. 8 A_1_), brain sections from vehicle-treated 5XFAD mice contained widespread GFAP staining (Fig. 8 A_2_), while 5XFAD animals treated with STX showed substantially reduced GFAP expression (Fig. 8 A_3_). Higher magnification views of GFAP staining in astrocytes are shown in Supplemental Fig. 3B and D. Quantification of this effect showed statistically significant differences in astrocytosis between groups within hippocampal regions (Fig. 8C; female: *χ*^*2*^ *=* 13.094, *p =* 0.014; male: *χ*^*2*^ *=* 11.843, *p =* 0.0027). In males, hippocampal astrocytosis was significantly reduced in STX-treated 5XFAD mice, compared with vehicle-treated animals (z = 2.4, *p* < 0.05) and a similar trend was seen in females (z = 1.9, *p =* 0.08). We also observed statistically significant differences in astrocytosis between groups within cortical regions (Fig. 8D; male: *χ*^*2*^ *=* 10.468, *p =* 0.0053; female: *χ*^*2*^ *=* 9.414, *p =* 0.0090), with more modest reductions associated with STX treatment in both males (z = 2.0, *p =* 0.07) and females (z = 0.8, *p =* 0.60). Likewise, when we labeled brain sections with biotinylated GSL (a marker for microglial activation) (Frautschy et al., 1998; Streit, 1990), we found that the extent of labeling was dramatically upregulated in 5XFAD mice, compared to vehicle-treated controls (Fig. 8 B_1_–B_2_), whereas STX treatment reduced overall labeling in 5XFAD brain sections (Fig. 8B_3_). Interestingly, the effects of STX on microglial activation were more pronounced in the cortex (Fig. 8F) than in hippocampal regions (Fig. 8E). In cortical regions, statistically significant associations in microgliosis between groups were detected in both females (*χ*^*2*^ *=* 11.741,=, *p =* 0.0028) and males (*χ*^*2*^ *=* 9.735,=, *p =* 0.0077), while in hippocampal regions, more moderate but significant group differences were also detected in females (*χ*^*2*^ *=* 9.211,=, *p =* 0.0100) and males (*χ*^*2*^ = 8.708,=, *p =* 0.129), although these effects did not reach statistical significance with the relatively small sample sizes use in this assay. As discussed below, these beneficial effects of STX on amyloid accumulation and associated reactive gliosis may be a consequence of improved mitochondrial function, similar to the effects of other compounds with mitoprotective activity.

## 4. Discussion

Although unacceptable side-effects preclude the use of estrogen-based therapies for patients at risk of AD (Dumanski et al., 2017; Manson et al., 2013; Shumaker et al., 2004), a variety of SERMs that target specific estrogen receptors (ER) have been shown to confer many of the neuroprotective benefits of E2 (Pandey et al., 2016; Wang et al., 2020; Zhao et al., 2013) suggesting that they might provide an alternative treatment strategy. However, compounds that engage either nuclear ER (ERα and ERβ) can also increase the risk of a variety of cancers (Coman et al., 2017; Liu et al., 2018; Tao et al., 2018) as well as other deleterious cellular responses, including mitochondrial dysfunction (Burstein et al., 2018; Colon-Caraballo, 2019). In contrast, STX is a novel non-steroidal ligand with unique properties: unlike conventional SERMs, STX is a highly selective agonist for the putative membrane estrogen receptor GqMER, with a 10^6^ reduced affinity (compared with estradiol) for ERα, ERβ, and the non-nuclear receptor GPER (Kelly and Ronnekleiv, 2015; Kenealy et al., 2011; Qiu et al., 2003). Although full characterization of GqMER is still in progress, extensive work has shown that activation of GqMER by STX induces rapid responses in CNS neurons via the heterotrimeric G protein Gαq (Qiu et al., 2006; Roepke et al., 2010; Smith et al., 2013). STX is orally bioavailable and has been found to match the neuroprotective effects of E2 in mouse models of ischemia, in part via activation of PI3K-Akt signaling (Inagaki and Etgen, 2013; Lebesgue et al., 2010). Equally important, STX does not induce estrogen-associated responses in peripheral tissues or promote cancer cell proliferation (Qiu et al., 2003; Qiu et al., 2006), suggesting that engagement of GqMER signaling by STX might provide a viable alternative for treating AD in both men and women.

In previous work, we showed that STX protected hippocampal neurons against Aβ neurotoxicity in vitro, mitigating the loss of mitochondrial function and synaptic integrity (Gray et al., 2016). We have now recapitulated these studies in the 5XFAD mouse model, demonstrating that orally administered STX attenuates both the mitotoxic and synaptotoxic effects of Aβ toxicity in vivo (Figs. 2–5). We also found that a two-month treatment with STX reduced key markers of astrocytosis and microglial activation (Fig. 8), suggesting an overall reduction in chronic inflammatory responses associated with AD (Gonzalez-Reyes et al., 2017; Leng and Edison, 2021). Lastly, the functional consequences of these neuroprotective effects were evident in moderately improved cognitive responses, whereby STX mitigated the decline in spatial memory performance without any adverse side effects associated with conventional estrogen replacement regimens in human subjects. Although STX produced only moderate effects on mouse behavior in this study, our encouraging results suggest this compound may have potential benefits in human at risk of AD when administered with optimized dosages and treatment regimens, particularly in females. The modest cognitive benefits (despite reduced amyloid levels) is also consistent with recent clinical trials of amyloid-lowering agents, which continue to be developed for important early disease-modifying effects, rather than purely symptomatic effects on behavior.

Interestingly, the neuroprotective effects of STX were generally more robust in females than in males. In part, these gender-specific effects may reflect the accelerated pathology that develops in 5XFAD females (compared to males of this line), reflecting the more widespread accumulation of Aβ in the brain (Bhattacharya et al., 2014; Maarouf et al., 2013; Oakley et al., 2006). Likewise, a correlation analysis identified positive relationships between MWM responses and several pathological markers that were significant or approached significance in females, while this relationship was less robust in males (Table 1). In this regard, the 5XFAD line provides an important model for investigating sex-based differences in response to candidate therapeutic compounds (Oblak et al., 2021). Accordingly, the lower Aβ burden reported in 5XFAD males might have muted the overall impact of STX treatments in males (compared to females), given the relatively small sample sizes used in this study (Maarouf et al., 2013; Oakley et al., 2006). Similarly, the fairly subtle reduction in behavioral responses that we observed in 5XFAD mice at 8 months is consistent with other recent studies using this model (Forner et al., 2021) and may have affected our ability to demonstrate a robust protective effect by STX. Alternatively, it is possible that receptors targeted by STX are more strongly expressed in females, although our experiments indicated similar trends for treatment effects in both males and females.

Unexpectedly, we also found that STX treatment reduced Aβ burden in animals treated from 6 to 8 months of life (Fig. 7A–C), during which amyloid pathology becomes increasingly pronounced in 5XFAD mice (Kimura and Ohno, 2009; Oakley et al., 2006). This effect was not anticipated, given that engagement of GqMER signaling by STX is not expected to alter APP synthesis or Aβ aggregation. One potential explanation is that STX might indirectly modulate Aβ production by supporting mitochondrial function, since elevated reactive oxygen species (ROS) levels resulting from mitochondrial dysfunction provoke amyloidogenic processing of APP, in part via enhanced beta secretase 1 (BACE1) and presenilin expression (Jo et al., 2010; Leuner et al., 2012; Tong et al., 2005). Alternatively, by supporting mitochondrial function, STX might also enhance the normal phagocytosis and clearance of pathogenic Aβ. This model is consistent with the effects of other candidate therapies that have Aβ-lowering effects in the absence of any direct effect on Aβ production (Logan et al., 2018; Matthews et al., 2019). In addition, we found that STX treatment reduced key markers of inflammation in the brain (including astrocytosis and microgliosis). In this regard, we postulate that STX might have therapeutic benefits independent of its effects on Aβ accumulation, providing an alternative to recent drug trials that have directly targeted amyloidosis in the brain with limited success.

## 5. Conclusions

In summary, these experiments indicate that STX has potential as a novel treatment for AD. By using a combination of methods to analyze markers for mitochondrial viability, synaptic integrity, brain inflammation, and surrogates of cognitive function in 5XFAD mice, we have shown that orally administered STX is both safe and neuroprotective in this well-characterized model of amyloid pathology. The beneficial effects of STX were generally more apparent in females than in males, potentially reflecting the accelerated course of amyloid pathology that occurs in female 5XFAD mice. Unexpectedly, STX treatment also reduced the accumulation of Aβ in the brain, possibly an indirect beneficial effect of improved mitochondrial function. In future studies, we will conduct a formal pre-clinical toxicological analysis and dose optimization in complementary models of both amyloid and tau pathology, with the goal of advancing STX towards early-stage clinical trials.

## Supplementary Material

1

2

3

## Figures and Tables

**Table 1:** Correlation between markers of neuropathology and cognitive responses in 5XFAD mice. For both females and males, we ran 84 correlations between six pathology variables, seven Morris Water Maze (MWM) test variables, seven Novel Object test variables. Because of violations of normality, we used Spearman correlations to assess the relationship between cognitive responses and neuropathology in these exploratory analyses (without alpha corrections). Correlations that were statistically significant, or approaching statistical significance, are indicated:

Significant Correlations in Females					
	GFAP Cortex (*N* = 25)	GSL Cortex (*N* = 23)	Abeta Plaque Cortex (*N* = 21)		GFAP Hippocampus (N = 25)
MWM 24-h Probe	−0.46^*^	−0.46^*^	−0.62^**^		−0.37^#^
MWM 72-h Probe			−0.41^#^		−0.41^*^

Significant Correlations in Males					

				Abeta Plaque Cortex (*N* = 12)	

MWM 72-h Probe				−0.56^#^		

# *p* = 0.06

* *p* < 0.05

** *p* < 0.01.

## Data Availability

Data will be made available on request.

## Abbreviations:

AD: Alzheimer’s Disease
Aβ: amyloid-beta (beta amyloid)
APP: Amyloid Precursor Protein
ATP5A: Nuclear-encoded ATP synthase F1 subunit alpha
BACE1: Beta secretase 1
E2: 17β-estradiol
ER: Estrogen receptor
ETC: Electron transport chain
FAD: Familial Alzheimer’s Disease
GAPDH: Glyceraldehyde-3phosphate dehydrogenase
GFAP: Glial Fibrillary Acidic Protein
GSL 1: *Griffonia simplicifolia* Lectin I
GqMER: G protein-coupled membrane estrogen receptor
Mt-ATP6: Mitochondrially encoded ATP synthase
MT-CO1: Mitochondrially encoded cytochrome *c* oxidase 1
Mt-CYB: Mitochondrially encoded cytochrome B
Mt-ND1: Mitochondrially encoded NADH dehydrogenase 1
MWM: Morris Water Maze
NDUFB8: Nuclear-encoded NADH dehydrogenase beta sub-complex subunit 8
PSD95: Post-synaptic density protein-95
qRT-PCR: quantitative reverse transcription polymerase chain reaction
ROS: Reactive oxygen species
SDHB: Nuclear-encoded Succinate dehydrogenase [ubiquinone] iron‑sulfur subunit
SERM: Selective estrogen receptor modulator
TBS: Tris-buffered saline
UQCR2: Mitochondrially encoded ubiquinol-cytochrome C reductase Core Protein 2
