# Supplementary figures and images for: The novel estrogen receptor modulator STX attenuates Amyloid-β neurotoxicity in the 5XFAD mouse model of Alzheimer’s disease
Source: Neurobiol Dis. Author manuscript; Available in PMC 2023 Apr 17. (PMC10108899; doi:10.1016/j.nbd.2022.105888)

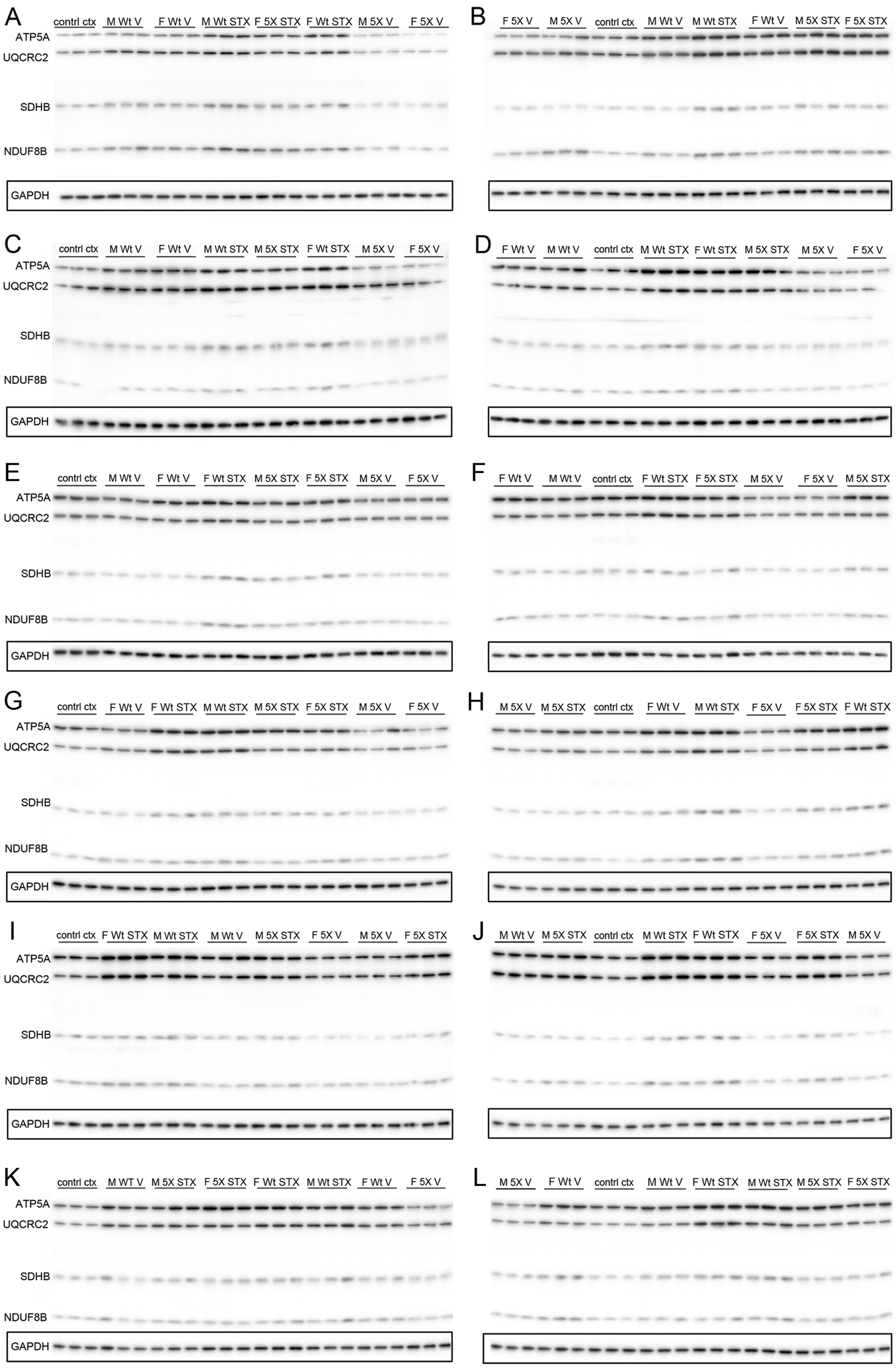

Supplement: 1 [file NIHMS1884282-supplement-1.jpg]

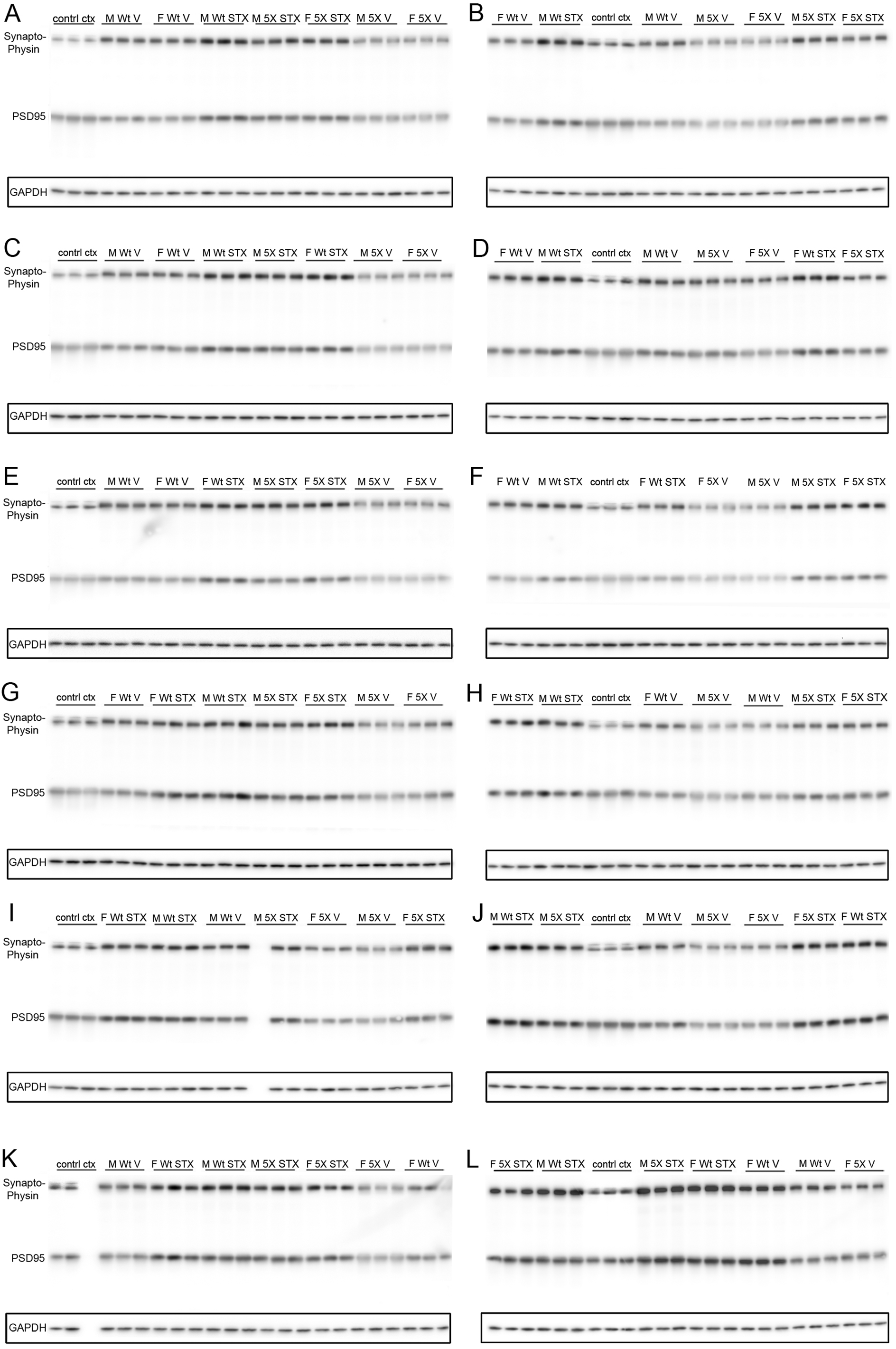

Supplement: 2 [file NIHMS1884282-supplement-2.jpg]

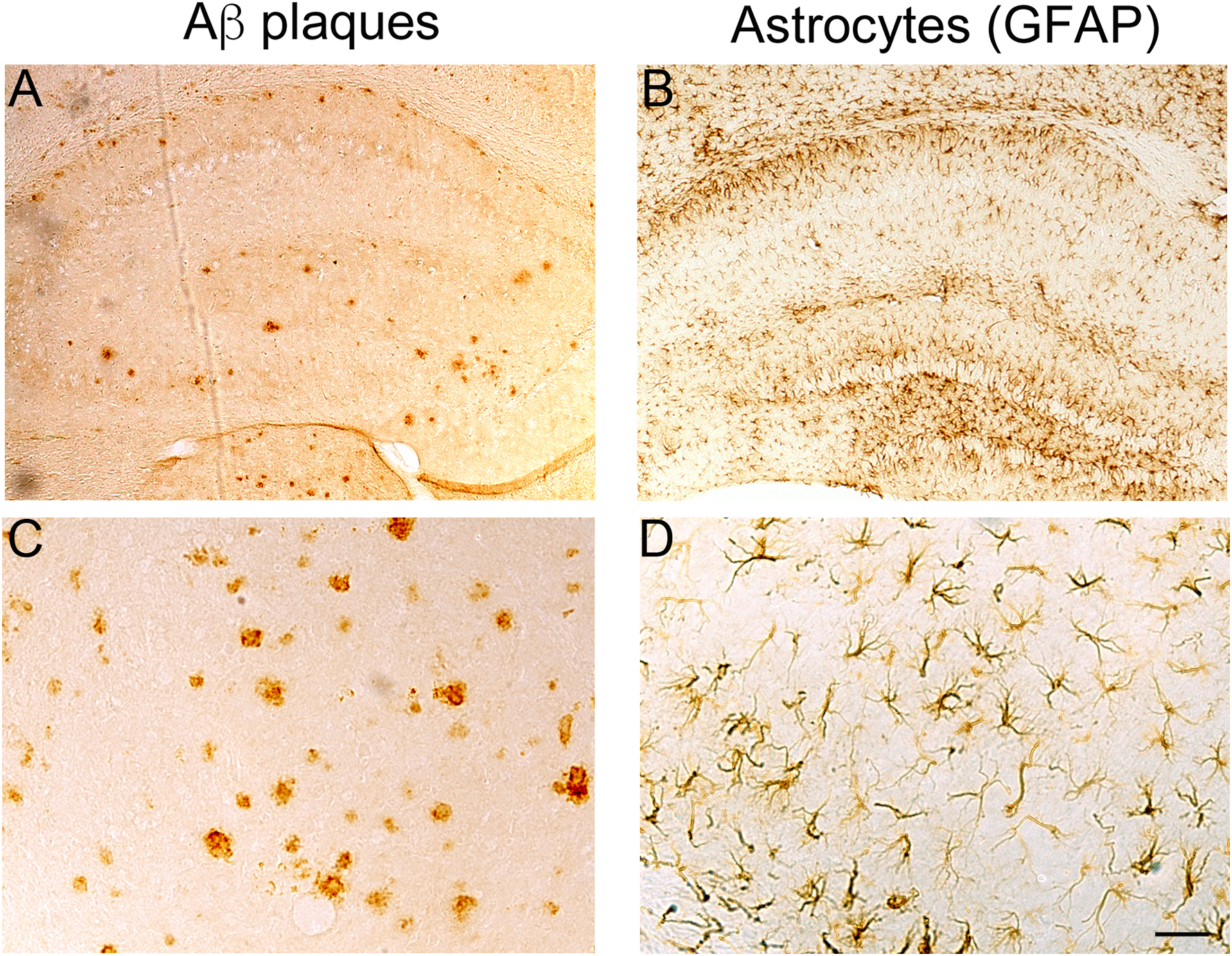

Supplement: 3 [file NIHMS1884282-supplement-3.jpg]
